# Characterization of Ten Novel Metabolites of a PAF Antagonist SY0916 in Rats Using LC-MS and NMR

**DOI:** 10.3390/metabo15040238

**Published:** 2025-03-31

**Authors:** Xin He, Tingting Zhang, Hongyi Zhao, Chen Ma

**Affiliations:** Institute of Materia Medica, Chinese Academy of Medical Science and Peking Union Medical College, Beijing 100050, China; hexin@imm.ac.cn (X.H.); zhangtingting-87@imm.ac.cn (T.Z.); zhaohongyicool@imm.ac.cn (H.Z.)

**Keywords:** SY0916, LC-HRMS, metabolites, NMR, metabolic profile

## Abstract

**Background:** SY0916 is a novel PAF receptor antagonist used to treat chronic immune-inflammatory diseases and is currently undergoing phase II clinical trials. However, SY0916 is rapidly transformed in vivo, suggesting a demand for metabolite screening. **Methods:** According to the similar MS fragmentation patterns of SY0916 and its five reported metabolites (M01, M02, M03, M05, and M06), a strategy based on two characteristic ions of *m/z* 170 and *m/z* 142 was employed to identify the potential metabolites in precursor screening in vivo, then LC-HRMS and NMR were applied to the metabolites characterization. **Results:** Two phase I metabolites (M07 and M08) were identified using LC-HRMS and NMR. Eight phase II metabolites, including six glutathione conjugates (M09-M14) and two sulfonate conjugates (M15 and M16), were identified using LC-HRMS. The possible metabolic pathways of SY0916 and fragmentation regularities of mass spectra of its metabolites were summarized. **Conclusions:** We preliminarily determined the metabolic profile of SY0916 in rats using LC-MS, providing insight into the metabolism of SY0916 in vivo and a basis for clinical studies and future applications.

## 1. Introduction

Rheumatoid arthritis (RA) is an autoimmune disease manifested by synovial inflammation, affecting approximately 1% of the global population and 1.7% of the elderly population [1,2,3]. Anti-inflammatory drugs have various adverse effects, such as gastric mucosal damage and immunosuppression [4,5], which calls for more rational treatment options to alleviate the pain in affected individuals. Platelet-activating factor (PAF) is originally an active lipid molecule produced by basophils binding to antigens in sensitized rabbits [6]. It participates in many inflammatory-related physiological regulations as a functional molecule [7,8]. Thus, the development of PAF receptor antagonists has significant physiological implications for the treatment of inflammatory diseases such as RA.

**Figure 1:**
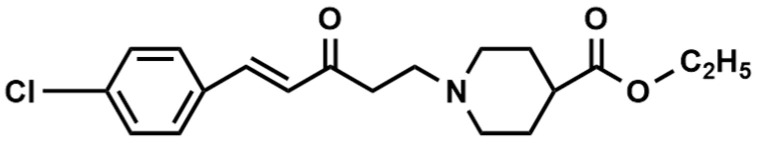
Chemical structure of SY0916.

SY0916 (Figure 1) is a novel PAF receptor antagonist. In preclinical research, SY0916 was shown to have a significant inhibitory effect on collagen-induced joint swelling in rats with RA and to affect bone metabolism by inhibiting osteoclast proliferation and modulating the RANKL-OPG-RANK axis [9]. Unlike other drugs prescribed for RA, SY0916 excels in providing gastric mucosal protection, making it suitable for long-term treatment [10].

SY0916 is stable in aqueous solution under acidic conditions [11]. Pei Hu et al. reported that SY0916 primarily metabolizes into five metabolites (M01, M02, M03, M05, and M06) in humans [12] via two types of metabolic reactions: reduction and hydrolysis. Meanwhile, SY0916 is eliminated quickly in humans and is present at low levels in biological samples such as plasma and urine, suggesting that a wide range of biotransformation reactions occur in vivo. Given the pharmacokinetic characteristics of low exposure, we believe that additional metabolites of SY0916 need to be discovered. The present study is aimed to elucidate the structures of new metabolites produced by SY0916. Ten new metabolites were identified in vivo based on the strategy of precursor scan using liquid chromatography–tandem mass spectrometry (LC-MS/MS) in rats, including phase I products (M07 and M08), glutathione conjugates (M09-M14) and sulfonate conjugates (M15 and M16). The structures of M07 and M08 were verified using nuclear magnetic resonance (NMR) and liquid chromatography–high-resolution mass spectrometry (LC-HRMS). These results provide a comprehensive understanding of the transformation of SY0916, and the metabolic profile also indicates the active sites in SY0916 in vivo, providing more valuable information for the research on drug safety and motivating the process of clinical experiments in humans.

## 2. Materials and Methods

### 2.1. Chemicals and Reagents

SY0916 was provided by the Institute of Materia Medica, Chinese Academy of Medical Sciences. HPLC-grade ammonium formate was obtained from CNW Technologies (Shanghai, China). Formic acid was gained from the MACKLIN reagent (Shanghai, China). HPLC-grade acetonitrile was gained from Honeywell Research Chemicals (Morris Plains, NJ, USA). Deionized water was purified from the Milli-Q water system (Bedford, MA, USA).

### 2.2. Instrumentation

The LC-HRMS system included the Dionex Ultimate^®^ 3000 RSLC liquid chromatography system and the Thermo Q Exactive Focus mass system (Thermo Scientific; Waltham, MA, USA) to determine the exact mass of each metabolite. The Shimpack XR-ODS II column (3.0 × 75 mm, 2.2 μm) (Shimadzu; Kyoto, Japan) was introduced at 35 °C for separation. Phases A and B in the mobile phase consisted of 10 mM ammonium formate buffer containing 0.1% formic acid and acetonitrile, respectively. The elution procedure was set as follows: 0 min, 10% B; 40 min, 90% B; 47 min, 90% B; 47.1 min, 10% B; 50 min, 10% B. The flow rate was set at 0.2 mL/min. The data acquisition process was carried out in positive ion mode, with optimized parameters including a spray voltage of 3300 V, capillary temperature of 320 °C, and probe heating temperature of 310 °C. The collision voltage was adjusted based on the sensitivity of all metabolites (Table S1). All LC-HRMS data were analyzed using Thermo Scientific Xcalibur 2.2 SP1 (Thermo Scientific; Waltham, MA, USA).

The LC-MS/MS system, including the Shimadzu UFLC XR and AB Sciex QTrap 5500 (AB Sciex; Framingham, MA, USA), was used to profile the metabolites in different biological samples. Analytical elution was performed as described in the LC-HRMS section. All mass parameters were set in positive ion mode and optimized as follows: source temperature, 550 °C; curtain gas, 35 psi; nebulizing gas, 50 psi; turbo source gas, 50 psi. Other voltages, such as the declustering potential, entrance potential, and collision energy, were modified to best suit each metabolite. All data were acquired and processed in Analyst 1.6.2 (AB Sciex; Framingham, MA, USA).

### 2.3. Animal Experiments

Wistar rats (male, 6–7 weeks, 200–220 g) were provided by Vital River Laboratory Animal Technology Co., Ltd. (Beijing, China) and grouped randomly for tissues and excretion collection, respectively. The rats were housed in animal chambers, where the ambient temperature and humidity were maintained within a relatively constant range. The animals were kept in a 12 h circadian rhythm. All experimental procedures were approved by the Animal Research Laboratory of the Chinese Academy of Medical Sciences and the Animal Ethics Review Board.

#### 2.3.1. Tissues Collection

At 6 h after the administration of SY0916 at 50 mg/kg, the rats were sacrificed, and tissues (including the liver, kidneys, stomach, and intestine) were collected and washed with saline. After drying with filter paper, the samples were homogenized in a solution (saline/formic acid 100:1, *v*/*v*) at a ratio of 1:3 (*w*/*v*). All samples were then frozen at −80 °C.

#### 2.3.2. Excretion Collection

To study the transformation of SY0916 in excretion, Wistar rats were administered SY0916 at a dose of 50 mg/kg. Urine and feces were collected in a 0–12 h period. A solution (saline/formic acid 100:1, *v*/*v*) was added at a ratio of 1:3 (*v*/*v*) for urine and 1:3 (*w*/*v*) for feces before homogenization. All excretion samples were frozen at −80 °C.

### 2.4. Sample Preparations

Volumes of 300 µL of tissue samples and 50 µL of urine and fecal samples were, respectively, added to 2 mL of ethyl acetate, vortexed for 3 min, and subjected to centrifugation at 7456× *g* for 10 min. The supernatant was then blown dry under a nitrogen flow at 40 °C, redissolved in 300 µL of acetonitrile, and filtered through 0.22 μm membranes. The injection volume was 3 µL for tissues and 1 µL for excretion samples.

### 2.5. Synthesis of M07 and M08

#### 2.5.1. Synthesis of M07

A solution of ethyl 1-(5-(4-chlorophenyl)-3-oxopentyl) piperidine-4-carboxylate hydrochloride (3.5 mmol) in dichloromethane (25 mL) was added to triethylamine (3.7 mmol) and allowed to react at room temperature for 1 h. To extract the mixture in dichloromethane, water was added to the system. The organic layer was then separated, washed with brine, and evaporated under a vacuum. A colorless oil was obtained as ethyl 1-(5-(4-chlorophenyl)-3-hydroxypentyl) piperidine-4-carboxylate (1.1 g, 93%).

A solution of the above oil (0.85 mmol) dissolved in ethyl acetate was mixed with an HCl/ethyl acetate solution (4 mol/L), followed by precipitation. After evaporation under vacuum, ethyl 1-(5-(4-chlorophenyl)-3-hydroxypentyl) piperidine-4-carboxylate hydrochloride (330 mg, 99%) was obtained as a white solid.

#### 2.5.2. Synthesis of M08

A mixture of ethyl 1-(5-(4-chlorophenyl)-3-hydroxypentyl) piperidine-4-carboxylate hydrochloride (0.59 mmol) and hydrochloric acid solution (1 mol/L, 9 mL) was reacted at 90 °C for 2 h. After evaporation under vacuum and freeze-drying, the final product of 1-(5-(4-chlorophenyl)-3-hydroxypentyl) piperidine-4-carboxylic acid hydrochloride was obtained as a white solid (210 mg, 99%).

## 3. Results and Discussion

### 3.1. Analytical Strategy Based on the MS Fragmentation Characteristics of Metabolites of SY0916

**Figure 2:**
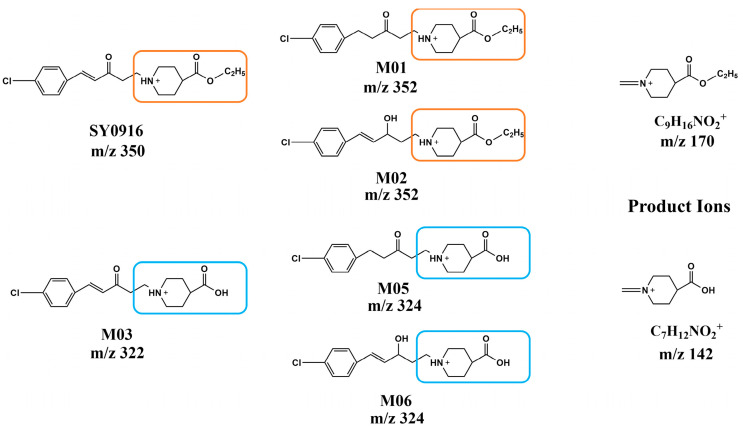
Protonated structures of SY0916 and five reported metabolites, with two characteristic product ions at *m/z* 142 and *m/z* 170.

Five metabolites of SY0916 (M01, M02, M03, M05, and M06) have been reported previously. SY0916 and its metabolites shared similar fragmentation patterns (Figure 2). The C-C bond next to the pyridine ring was broken by α—cleavage; the fragment at *m*/*z* 170 was the characteristic ion of SY0916, M01, and M02, and the ion at *m*/*z* 142 was the characteristic ion of M03, M05, and M06 [13]. The consistent characteristics of the fragment ions of SY0916 and the five reported metabolites provided the basis for an analytical strategy of targeted product ions to excavate new metabolites of SY0916. Based on the strategy of the precursor scan, ten new metabolites (M07-M16) were excavated; the chromatography of these metabolites is shown in Figure S1, while the fragments data and elemental composition of ten new metabolites are shown in Table S2.

LC-MS/MS analysis was utilized to detect SY0916 and its metabolites in tissues and excretion samples. The metabolites detected in different sample types are displayed in Table S3. M01 and M11-M14 were specifically located in the stomach, whereas M09 and M10 were detected in both the liver and stomach. M03 was detected in the stomach, intestine, feces, and urine samples. M05 and M06 were observed in all samples. M07 was mainly detected in the feces, whereas M08 was detected in the kidneys, feces, and urine. M15 was detected in the liver, stomach, intestine, and feces, whereas M16 was observed in all samples.

### 3.2. Characterization of Ten New Metabolites of SY0916

#### 3.2.1. Phase I Metabolites

Two reductive metabolites, M07 and M08, were identified through an analytical strategy in the precursor scan, and their structures were characterized using LC-HRMS and ^1^H NMR. Given the importance of phase I metabolites in drug metabolism, M07 and M08 may have similar bioactivities to those of SY0916. Therefore, they were chemically synthesized to confirm the structures.

##### HRMS and ^1^H NMR Verification of M07

**Figure 3:**
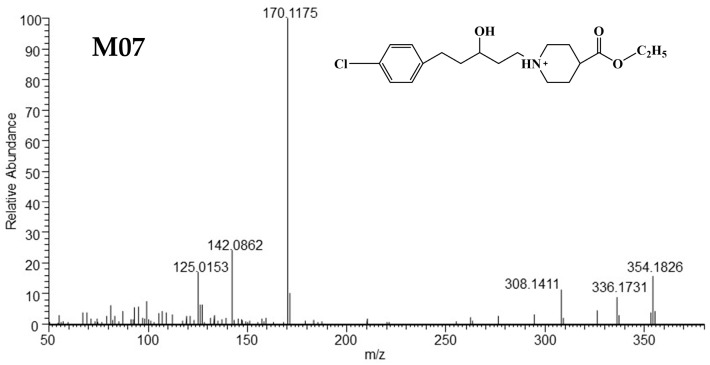
The HRMS/MS spectra and supposed structure of M07.

The [M+H]^+^ of M07 at m/z 354.1826 was consistent with the elemental composition of C_19_H_29_O_3_NCl^+^, exhibiting an increase of 4 Da (4H) compared to SY0916. The dominant fragment ion at *m/z* 170.1175 was the same as that for SY0916 [13]. The quasi-molecular ion of M07 lost H_2_O and formed a fragment ion at *m/z* 336.1731. Based on the above results, the possible structure of M07 is a reduction product of the C=C and the C=O groups in SY0916. The HRMS/MS spectrum and structure of M07 are shown in Figure 3, and the possible fragmentation pathway of M07 is plotted in Figure S2.

The ^1^H NMR (400 MHz, DMSO-d_6_) of M07 was as follows: δ ppm: 10.02 (s, 1H), 7.33 (d, *J* = 8.8 Hz, 2H), 7.24 (d, *J* = 8.0 Hz, 2H), 4.90 (d, *J* = 5.6 Hz, 1H), 4.09 (q, *J* = 7.2 Hz, 2H), 3.53–3.41 (m, 3H), 3.19–2.54 (m, 7H), 2.09–1.58 (m, 8H), 1.90 (t, *J* = 7.2 Hz, 3H). The ^1^H NMR spectra and H assignments are attached in Table S4.

##### HRMS and ^1^H NMR Verification of M08

**Figure 4:**
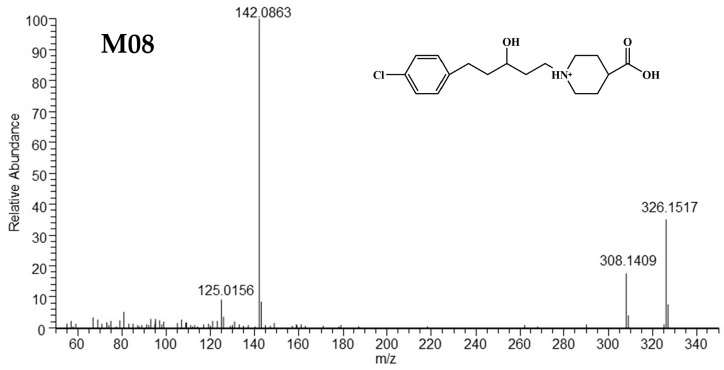
The HRMS/MS spectra and supposed structure of M08.

The [M+H]^+^ of M08 was located at *m/z* 326.1517 (C_17_H_25_O_3_NCl^+^), which exhibited an increase of 4 Da (4H) than that of M03 (C_17_H_21_O_3_NCl^+^). The abundant fragment ion at *m/z* 142.0863 was identical to that for M03 [13]. The [M+H]^+^ removed H_2_O to generate a fragment ion at *m/z* 308.1409. These data suggest that M08 was generated by the reduction in the C=C and the C=O groups in M03. The ion at *m/z* 125.0156 was formed by the loss of C_10_H_19_O_3_N from [M+H]^+^. The HRMS spectra and predicted structure of M08 are shown in Figure 4, and the fragmentation pathway of M08 is shown in Figure S3.

The ^1^H NMR (400 MHz, DMSO-d_6_) of M08 was as follows: δ ppm: 12.54 (brs, 1H), 10.43 (brs, 1H), 7.33 (d, *J* = 7.6 Hz, 2H), 7.24 (d, *J* = 8.0 Hz, 2H), 4.91 (s, 1H), 3.55–2.55 (m, 10H), 2.09–1.58 (m, 8H). The ^1^H NMR spectra and assignments of the H atom are concluded in Table S4.

#### 3.2.2. Glutathione Conjugates

Six glutathione conjugates (M09-M14) were detected in the stomach and liver and then identified using LC-HRMS to elucidate their structures. The isomers of M09 and M10, as well as M12 and M13, were bound to SY0916 and M03, respectively. M11 and M14 were determined to be glutathione conjugates originating from M02 and M06, respectively.

##### Mass Spectrometric Characteristics of M09 and M10

**Figure 5:**
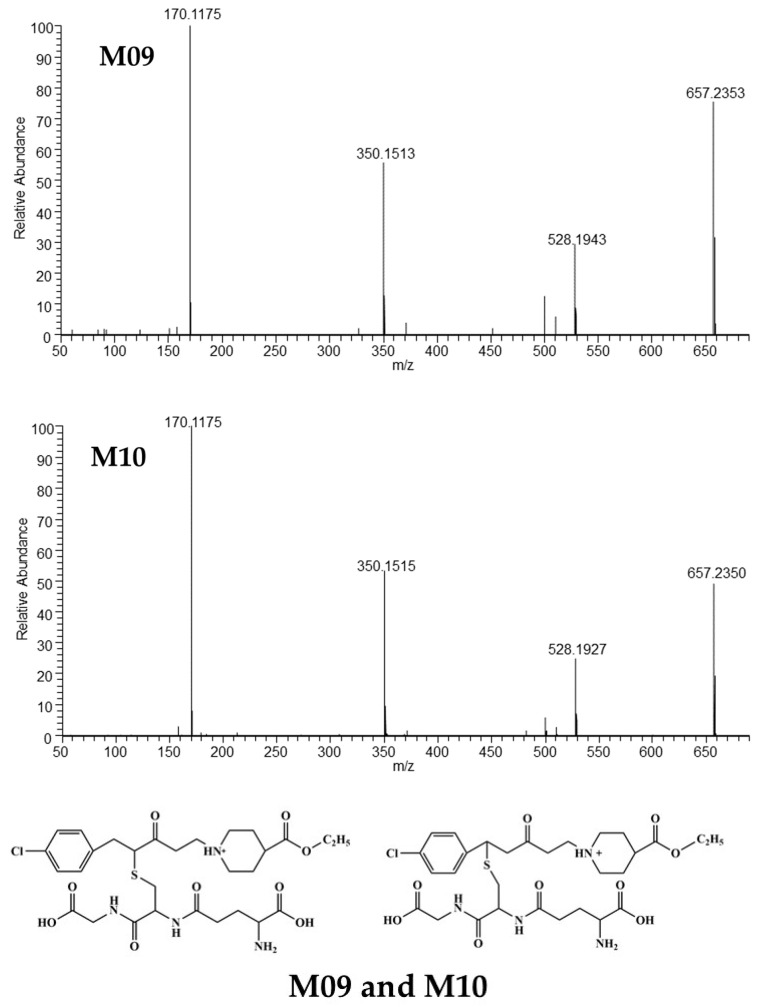
The HRMS spectra and speculative structures of M09 and M10.

M09 and M10 were isomers with the same elemental composition (C_29_H_42_O_9_N_4_ClS^+^) and HRMS/MS spectra (Figure 5). The [M+H]^+^ of M09 was observed at *m/z* 657.2353 (C_29_H_42_O_9_N_4_ClS^+^), which was 307 Da (C_10_H_17_O_6_N_3_S) more than SY0916. The fragment ion at *m/z* 528.1943 was reduced by 129 Da (-C_5_H_7_NO_3_) when compared with [M+H]^+^, which was a symbol of glutathione adducts by the breakage of the amide bond in glutathione and lost γ-glutamyl [14]. The fragment ions at *m/z* 350.1513 and *m/z* 170.1175 were the same as those of SY0916, suggesting that there was the same structure as SY0916. Based on the above, M09 and M10 are glutathione adducts conjugated on the olefinic bond because of the high activity between glutathione and the Michael acceptor [15]. However, the binding position of the glutathione was not determined. The speculated cleavage pathways of M09 and M10 are shown in Figure S4.

##### Mass Spectrometric Characteristics of M11

**Figure 6:**
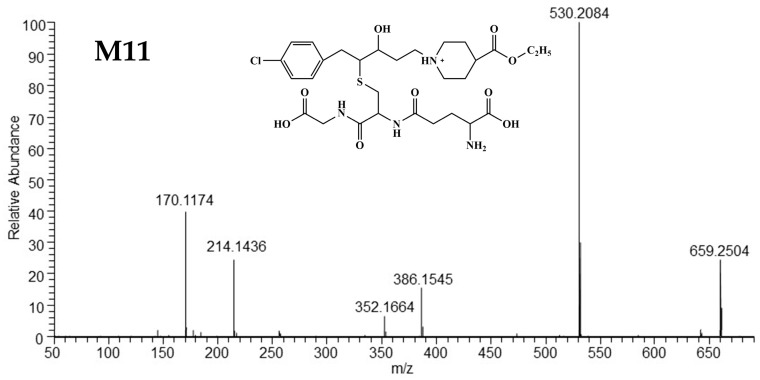
The mass spectra and supposed structures of M11.

The protonated molecular ion of M11 was exhibited at *m/z* 659.2504 (C_29_H_44_O_9_N_4_ClS^+^). The abundant ion at *m/z* 530.2084 was formed through a loss of γ-glutamyl (129 Da) from [M+H]^+^, which signified the symbol of glutathione adducts. Characterized ions at *m/z* 352.1664 and *m/z* 170.1174 were identical to those of M02 [13], suggesting M02 was conjugated to glutathione. The predicted structure and HRMS spectra of M11 are shown in Figure 6, and the fragmentation cleavage is plotted in Figure S5.

##### Mass Spectrometric Characteristics of M12 and M13

**Figure 7:**
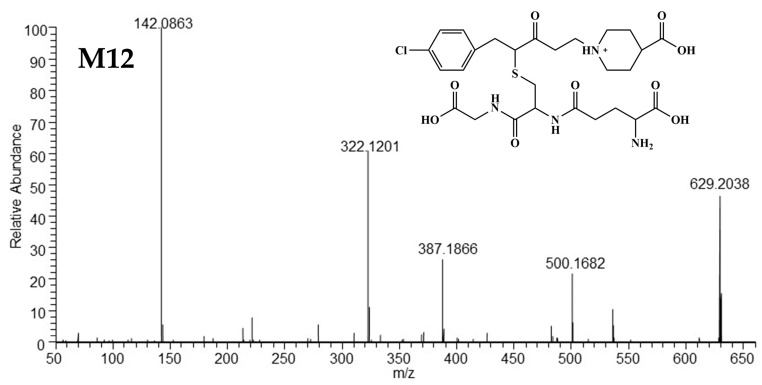
The HRMS spectra and predicted structures of M12.

**Figure 8:**
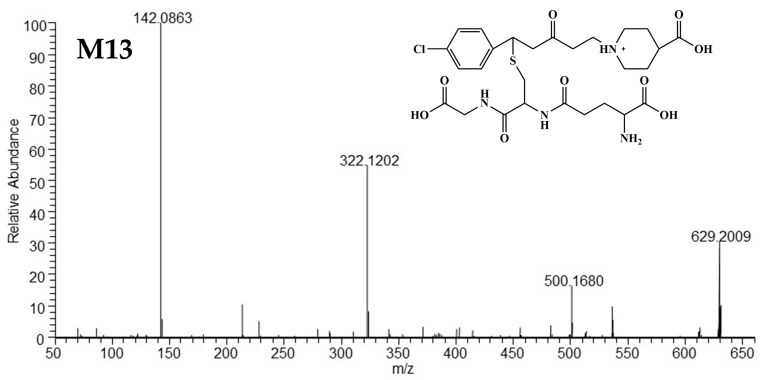
The HRMS spectra and predicted structures of M13.

The [M+H]^+^ of the isomers M12 and M13 was located at *m/z* 629 (C_27_H_38_O_9_N_4_ClS^+^) and generated the same fragment ions at *m/z* 500, *m/z* 322, and *m/z* 142, sharing similar HRMS spectra with those of M09 and M10 (Figure 5). The two main ions at *m/z* 322 and *m/z* 142 were typical of M03. Ion at *m/z* 500 was generated from the loss of γ-glutamyl (129 Da) from [M+H]^+^. These findings indicate that M12 and M13 are glutathione adducts bound to M03 and conjugated via the olefinic bond. Ion at *m/z* 387.1866 was observed only in M12. The protonated molecular ion of M12 can distinctly lose the 4-chlorotoluene moiety, glycine fragment (breakage of the amide bond [16]), and carboxyl moiety to form the ion at *m/z* 387.1866, which differs from M13 and shares the position of glutathione binding on the C=C bond of M03. The HRMS/MS spectra and predicted structures of M12 and M13 are displayed in Figure 7 and Figure 8, and the corresponding fragmentation pathways of M12 and M13 are detailed in Figures S6 and S7.

##### Mass Spectrometric Characteristics of M14

**Figure 9:**
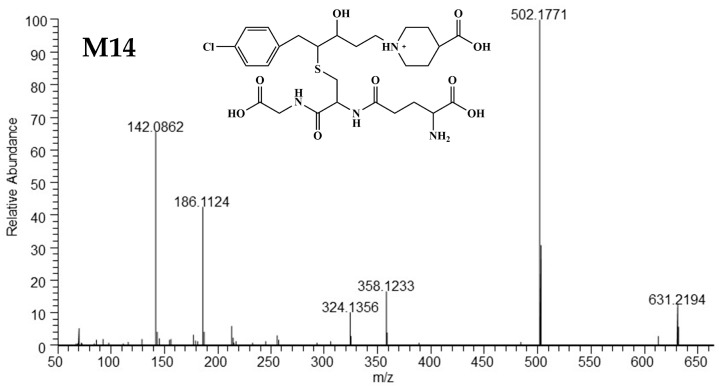
The high-resolution mass spectra and structure of M14.

The [M+H]^+^ of M14 was exhibited at *m/z* 631.2194 (C_27_H_40_O_9_N_4_ClS^+^), and the HRMS/MS spectra and structure are displayed in Figure 9. The ions at *m/z* 631.2194, *m/z* 502.1771, *m/z* 358.1233, *m/z* 324.1356, *m/z* 186.1124, *m/z* 142.0862 showed a decrease of 28 Da (C_2_H_4_) compared with the fragment ions in M11, suggesting M14 may arise from the hydrolysis of the ethyl ester group in M11. The detailed fragmentation mechanism is illustrated in Figure S8.

#### 3.2.3. Sulphonate Conjugates

Two sulfonate conjugates (M15 and M16) were mainly detected in tissues (the liver, intestine, and stomach) and excretion when screening through LC-MS/MS. Given their mass characteristics, M15 and M16 could be sulfonate conjugates of SY0916 and M03, respectively.

##### Mass Spectrometric Characteristics of M15

**Figure 10:**
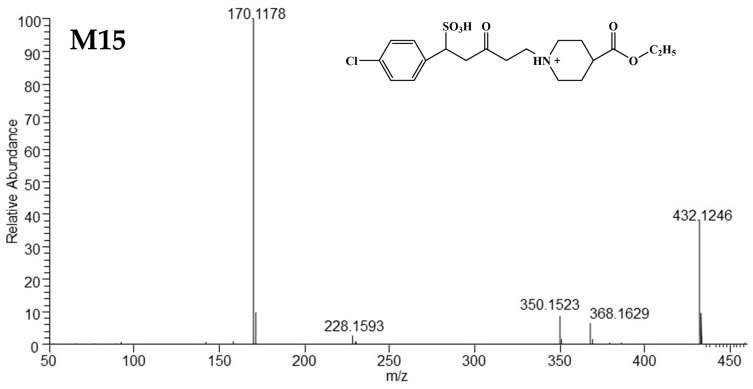
The HRMS spectra and predicted structure of M15.

M15 provided the [M+H]^+^ at *m*/*z* 432.1246 (C_19_H_27_O_6_NClS^+^), with a mass shift by +82 Da relative to SY0916, indicating the addition of H_2_SO_3_ to the element composition. The fragment ions at m/z 350.1523 and *m*/*z* 170.1178 were identical to SY0916. The [M+H]^+^ of M15 lost SO_2_ and formed an ion at *m*/*z* 368.1629. The ion at m/z 228.1593 arose from [M+H]^+^ after losing -C_2_H_5_O_3_ClS, indicating the binding site of H_2_SO_3_ was next to the side of 4-chlorophenyl on the C=C bond of SY0916. These data indicated that the structure of M15 was likely a sulphonate conjugate derived from SY0916. The mass spectra of M15 are detailed in Figure 10, and a possible fragmentation pathway is shown in Figure S9.

##### Mass Spectrometric Characteristics of M16

**Figure 11:**
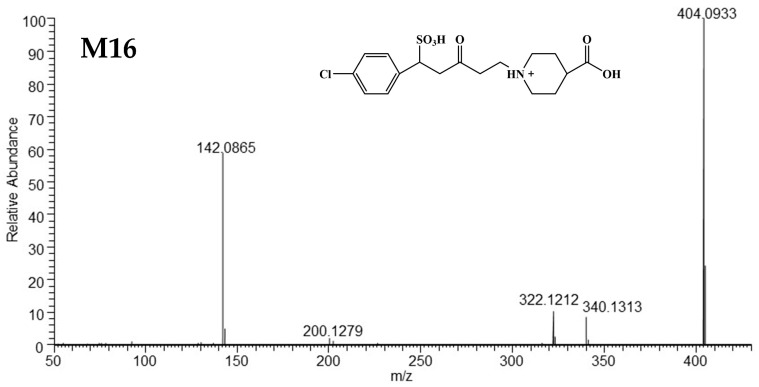
The HRMS spectra and proposed structure of M16.

The quasi-molecular ion of M16 at *m/z* 404.0933 (C_17_H_23_O_6_NClS^+^) was shifted by +82 Da (H_2_SO_3_) compared with that of M03. The characteristic ions at *m/z* 322.1212 and *m/z* 142.0865 were the same as those in M03, indicating that M03 may be the precursor that binds to sulfonic acid to generate M16. Ions at *m/z* 340.1313 and *m/z* 200.1279 showed a decrease of 28 Da (C_2_H_4_) compared with fragment ions in M15, corresponding to the same binding site on the C=C bond of M03. The HRMS/MS spectrum and postulated structure are shown in Figure 11, and detailed information on the product fragments is shown in Figure S10.

### 3.3. Transformation of SY0916 In Vivo

**Figure 12:**
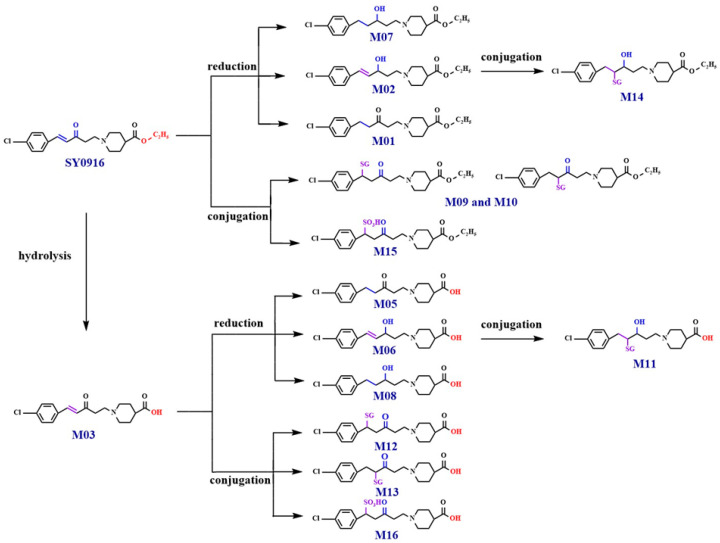
The possible metabolic pathways of SY0916.

SY0916 is highly reactive at the sites of the carbonyl and olefinic bond. The phase I transformation of SY0916 tended to involve the reduction in the C=C and C=O bond (M01, M02, and M07), whereas the hydrolysis of ethyl triggered the generation of M03. M05, M06, and M08 were formed by the reduction in the C=C and C=O groups of M03. In addition, conjugation reactions of the C=C bond in SY0916, M02, M03, and M06 were potent. M09-M14 were the products of glutathione conjugation, whereas M15 and M16 were identified as sulfonate conjugates. The possible metabolic pathways of SY0916 are summarized in Figure 12.

The Guidance of Metabolites in Safety Testing [17] has prompted increased awareness of the potential impacts of drug metabolism on safety and efficacy [18]. Some phase I metabolites have the potential for higher bioactivity than that of the original drug substance, whereas the transformation of phase II metabolites is related to chemical detoxification [19]. The characterization of new metabolites enriched the metabolite profile of SY0916. To better understand how the ten new metabolites affect the therapeutic effects of SY0916, bioactivity screening and pharmacokinetic experiments are needed in further research to provide a basis for clinical applications.

## 4. Conclusions

Ten new metabolites of SY0916 were excavated through a precursor scan strategy and characterized using LC-HRMS and NMR, expanding the metabolic profile beyond the five reported metabolites. Among the novel metabolites, two phase I metabolites were synthesized and verified through the ^1^H NMR spectrum.

SY0916 was detected in all types of biological samples (the liver, kidneys, stomach, intestine, feces, and urine), suggesting the potential for extensive transformation in vivo. The metabolism of SY0916 contained Phase I reactions (reduction and hydrolysis) and Phase II reactions (glutathione conjugation and sulfonate conjugation).

This study provides insight into the metabolism of SY0916 in rats. The metabolic profile is expected to guide the clinical application of SY0916 and its derivatives; further research on the pharmacokinetic characteristics of the newly identified metabolites will improve our understanding of their potential with respect to drug efficacy and safety.

## Data Availability

The data in this study are available in this article.

## Supplementary Materials

The following supporting information can be downloaded at: https://www.mdpi.com/article/10.3390/metabo15040238/s1, Figure S1: the chromatography of 10 new metabolites of SY0916; Figure S2: the possible fragmentation pathway of M07 in HRMS/MS spectrum; Figure S3: the possible fragmentation pathway of M08 in HRMS/MS spectrum; Figure S4: the possible fragmentation pathway of M09 and M10 in HRMS/MS spectrum; Figure S5: the possible fragmentation pathway of M11 in HRMS/MS spectrum; Figure S6: the possible fragmentation pathway of M12 in HRMS/MS spectrum; Figure S7: the possible fragmentation pathway of M13 in HRMS/MS spectrum; Figure S8: the possible fragmentation pathway of M14 in HRMS/MS spectrum; Figure S9: the possible fragmentation pathway of M15 in HRMS/MS spectrum; Figure S10: the possible fragmentation pathway of M16 in HRMS/MS spectrum; Table S1: optimized parameters of novel metabolites of SY0916 in high-resolution mass; Table S2: the ions and elemental composition of 10 metabolites; Table S3: the detection of SY0916 and its metabolites in different samples; Table S4: the H assignments and ^1^H NMR spectrum of M07 and M08.
