# Supplementary material for: Characterization of Ten Novel Metabolites of a PAF Antagonist SY0916 in Rats Using LC-MS and NMR
Source: Metabolites. 2025 Mar 31;15(4):238. doi: 10.3390/metabo15040238 (PMC12029730; doi:10.3390/metabo15040238)
Supplement: Supplementary file 1 [file metabolites-15-00238-s001.zip › supplementary files/Table S.doc]

**Captions**

Table S1 Optimized parameters of novel metabolites of SY0916 in HRMS.

Table S2 the ions and elemental composition of 10 metabolites.

Table S3 the detection of SY0916 and its metabolites in different samples.

Table S4 the H assignments and ^1^H NMR spectrum of M07 and M08.

Table S1. Optimized parameters of novel metabolites of SY0916 in high-resolution mass

| **Metabolic**  **Classification** | **ID** | **Collision Energy,**  **V** |
| --- | --- | --- |
| Phase I Metabolites | M07 | 30 |
|  | M08 | 25 |
| Phase II Metabolites | M09 | 20 |
|  | M10 |  |
|  | M11 | 30 |
|  | M12 | 20 |
|  | M13 |  |
|  | M14 | 30 |
|  | M15 | 20 |
|  | M16 |  |

Table S2. The ions and elemental composition of 10 metabolites

| **Metabolic**  **Classification** | **ID** | **Observed**  ***m/z*** | **Predicted**  ***m/z*** | **Elemental**  **Composition** | **Mass**  **Error,**  **Δppm** |
| --- | --- | --- | --- | --- | --- |
| Phase I metabolites | M07 | 354.1826 | 354.1831 | C_19_ H_29_ O_3_ N Cl^+^ | -1.412 |
|  |  | 336.1731 | 336.1725 | C_19_ H_27_ O_2_ N Cl^+^ | 1.785 |
|  |  | 308.1411 | 308.1412 | C_17_ H_23_ O_2_ N Cl^+^ | -0.325 |
|  |  | 170.1175 | 170.1176 | C_9_ H_16_ O_2_ N^+^ | -0.588 |
|  |  | 142.0862 | 142.0863 | C_7_ H_12_ O_2_ N^+^ | -0.704 |
|  |  | 125.0153 | 125.0153 | C_7_ H_6_ Cl^+^ | 0.000 |
|  | M08 | 326.1517 | 326.1518 | C_17_ H_25_ O_3_ N Cl^+^ | -0.307 |
|  |  | 308.1409 | 308.1412 | C_17_ H_23_ O_2_ N Cl^+^ | -0.974 |
|  |  | 142.0863 | 142.0863 | C_7_ H_12_ O_2_ N^+^ | 0.000 |
|  |  | 125.0156 | 125.0153 | C_7_ H_6_ Cl^+^ | 2.400 |
| Phase II metabolites | M09 | 657.2353 | 657.2356 | C_29_ H_42_ O_9_ N_4_ Cl S^+^ | -0.456 |
|  |  | 528.1943 | 528.1930 | C_24_ H_35_ O_6_ N_3_ Cl S^+^ | 2.461 |
|  |  | 350.1513 | 350.1518 | C_19_ H_25_ O_3_ N Cl ^+^ | -1.428 |
|  |  | 170.1175 | 170.1176 | C_9_ H_16_ O_2_ N^+^ | -0.588 |
|  | M10 | 657.2350 | 657.2356 | C_29_ H_42_ O_9_ N_4_ Cl S^+^ | -0.913 |
|  |  | 528.1927 | 528.1930 | C_24_ H_35_ O_6_ N_3_ Cl S^+^ | -0.568 |
|  |  | 350.1515 | 350.1518 | C_19_ H_25_ O_3_ N Cl^+^ | -0.857 |
|  |  | 170.1175 | 170.1176 | C_9_ H_16_ O_2_ N^+^ | -0.588 |
|  | M11 | 659.2504 | 659.2512 | C_29_ H_44_ O_9_ N_4_ Cl S^+^ | -1.213 |
|  |  | 530.2084 | 530.2086 | C_24_ H_37_ O_6_ N_3_ Cl S^+^ | -0.377 |
|  |  | 386.1545 | 386.1551 | C_19_ H_29_ O_3_ N Cl S^+^ | -1.554 |
|  |  | 352.1664 | 352.1674 | C_19_ H_27_ O_3_ N Cl^+^ | -2.840 |
|  |  | 214.1436 | 214.1438 | C_11_ H_20_ O_3_ N^+^ | -0.934 |
|  |  | 170.1174 | 170.1176 | C_9_ H_16_ O_2_ N^+^ | -1.176 |
|  | M12 | 629.2038 | 629.2042 | C_27_ H_38_ O_9_ N_4_ Cl S^+^ | -0.636 |
|  |  | 500.1682 | 500.1617 | C_22_ H_31_ O_6_ N_3_ Cl S^+^ | 12.996 |
|  |  | 387.1866 | 387.1828 | C_17_ H_29_O_5_N_3_S^+^ | 9.841 |
|  |  | 322.1201 | 322.1204 | C_17_ H_21_ O_3_ N Cl^+^ | -0.931 |
|  |  | 142.0863 | 142.0863 | C_7_ H_12_ O_2_ N^+^ | 0 |
|  | M13 | 629.2009 | 629.2042 | C_27_ H_38_ O_9_ N_4_ ClS^+^ | -5.245 |
|  |  | 500.1680 | 500.1617 | C_22_ H_31_ O_6_ N_3_ Cl S^+^ | 12.596 |
|  |  | 322.1202 | 322.1204 | C_17_ H_21_ O_3_ N Cl^+^ | -0.621 |
|  |  | 142.0863 | 142.0863 | C_7_ H_12_ O_2_ N^+^ | 0 |
|  | M14 | 631.2194 | 631.2199 | C_27_ H_40_ O_9_ N_4_ ClS^+^ | -0.792 |
|  |  | 502.1771 | 502.1773 | C_22_ H_33_ O_6_ N_3_ Cl S^+^ | -0.398 |
|  |  | 358.1233 | 358.1238 | C_17_ H_25_ O_3_ N Cl S^+^ | -1.396 |
|  |  | 324.1356 | 324.1361 | C_17_ H_23_ O_3_ N Cl^+^ | -1.543 |
|  |  | 186.1124 | 186.1125 | C_9_ H_16_ O_3_ N^+^ | -0.537 |
|  |  | 142.0862 | 142.0863 | C_7_ H_12_ O_2_ N^+^ | -0.704 |
|  | M15 | 432.1246 | 432.1242 | C_19_ H_27_ O_6_ N Cl S^+^ | 0.926 |
|  |  | 368.1629 | 368.1623 | C_19_ H_27_ O_4_ N Cl^+^ | 1.630 |
|  |  | 350.1523 | 350.1518 | C_19_ H_25_ O_3_ N Cl^+^ | 1.428 |
|  |  | 228.1593 | 228.1594 | C_12_ H_22_ O_3_ N^+^ | -0.438 |
|  |  | 170.1178 | 170.1176 | C_9_ H_16_ O_2_ N^+^ | 1.176 |
|  | M16 | 404.0933 | 404.0929 | C_17_ H_23_ O_6_ N Cl S^+^ | 0.990 |
|  |  | 340.1313 | 340.1310 | C_17_ H_23_ O_4_ N Cl^+^ | 0.882 |
|  |  | 322.1212 | 322.1205 | C_17_ H_21_ O_3_ N Cl^+^ | 2.173 |
|  |  | 200.1279 | 200.1281 | C_10_ H_18_ O_3_ N^+^ | -0.999 |
|  |  | 142.0865 | 142.0863 | C_7_ H_12_ O_2_ N^+^ | 1.408 |

Table S3. the detection of SY0916 and its metabolites in different samples

|  | **Liver** | **Kidney** | **Stomach** | **Intestine** | **Feces** | **Urine** |
| --- | --- | --- | --- | --- | --- | --- |
| **SY0916** | **＋** | **＋** | **＋** | **＋** | **＋** | **＋** |
| **M01** | **－** | **－** | **＋** | **－** | **－** | **－** |
| **M02** | **－** | **－** | **－** | **－** | **－** | **－** |
| **M03** | **－** | **－** | **＋** | **＋** | **＋** | **＋** |
| **M05** | **＋** | **＋** | **＋** | **＋** | **＋** | **＋** |
| **M06** | **＋** | **＋** | **＋** | **＋** | **＋** | **＋** |
| **M07** | **－** | **－** | **－** | **－** | **＋** | **－** |
| **M08** | **－** | **＋** | **－** | **－** | **＋** | **＋** |
| **M09** | **＋** | **－** | **＋** | **－** | **－** | **－** |
| **M10** | **＋** | **－** | **＋** | **－** | **－** | **－** |
| **M11** | **－** | **－** | **＋** | **－** | **－** | **－** |
| **M12** | **－** | **－** | **＋** | **－** | **－** | **－** |
| **M13** | **－** | **－** | **＋** | **－** | **－** | **－** |
| **M14** | **－** | **－** | **＋** | **－** | **－** | **－** |
| **M15** | **＋** | **－** | **＋** | **＋** | **＋** | **－** |
| **M16** | **＋** | **＋** | **＋** | **＋** | **＋** | **＋** |

＋: detected in corresponding samples

－: not detected in corresponding samples, lower than the limit of detection

Table S4. The H assignments and ^1^H NMR spectrum of M07 and M08

| **Metabolite ID** | |
| --- | --- |
|   **M07** | |
| **Assignment** | δ_H_ (ppm) |
| **2, 6** | 7.24 (d, J = 8.0 Hz, 2H) |
| **3, 5** | 7.33 (d, J = 8.8 Hz, 2H) |
| **7, 9** | 3.53-3.41 (m, 3H) |
| **8, 10, 15, 17** | 2.09-1.58 (m, 8H) |
| **11** | 4.90 (d, J = 5.6 Hz, 1H) |
| **12, 14, 16, 18** | 3.19-2.54 (m, 7H) |
| **20** | 4.09 (q, J = 7.2 Hz, 2H) |
| **21** | 1.90 (t, J = 7.2 Hz, 3H) |
| **22** | 10.02 (s, 1H) |

| **Metabolite ID** | |
| --- | --- |
|   **M08** | |
| **Assignment** | δ_H_ (ppm) |
| **2, 6** | 7.24 (d, J = 8.0 Hz, 2H) |
| **3, 5** | 7.33 (d, J = 7.6 Hz, 2H) |
| **7, 9, 12, 14, 16, 18** | 3.55-2.55 (m, 10H) |
| **8, 10, 15, 17** | 2.09-1.58 (m, 8H) |
| **11** | 4.91 (s, 1H) |
| **20** | 12.54 (brs, 1H) |
| **21** | 10.43 (brs, 1H) |

DMSO-d_6_ was used as the standard NMR solvents.

All data for ^1^H NMR was processed on Quantum-1 400MHz (Q.One Instruments Ltd; Wuhan, China)
