# Supplementary material for: Characterization of Ten Novel Metabolites of a PAF Antagonist SY0916 in Rats Using LC-MS and NMR
Source: Metabolites. 2025 Mar 31;15(4):238. doi: 10.3390/metabo15040238 (PMC12029730; doi:10.3390/metabo15040238)
Supplement: Supplementary file 1 [file metabolites-15-00238-s001.zip › supplementary files/Figure S-review2.doc]

**Captions**

Figure S1 the chromatography of 10 new metabolites of SY0916.

Figure S2 the possible fragmentation pathway of M07 in HRMS/MS spectrum.

Figure S3 the possible fragmentation pathway of M08 in HRMS/MS spectrum.

Figure S4 the possible fragmentation pathway of M09 and M10 in HRMS/MS spectrum.

Figure S5 the possible fragmentation pathway of M11 in HRMS/MS spectrum.

Figure S6 the possible fragmentation pathway of M12 in HRMS/MS spectrum.

Figure S7 the possible fragmentation pathway of M13 in HRMS/MS spectrum.

Figure S8 the possible fragmentation pathway of M14 in HRMS/MS spectrum.

Figure S9 the possible fragmentation pathway of M15 in HRMS/MS spectrum.

Figure S10 the possible fragmentation pathway of M16 in HRMS/MS spectrum.

Figure S1	Chromatography of 10 new metabolites

Figure S2	the possible fragmentation pathway of M07 in HRMS/MS spectrum

Figure S3	the possible fragmentation pathway of M08 in HRMS/MS spectrum

Figure S4	the possible fragmentation pathway of M09 and M10 in HRMS/MS spectrum

Figure S5	the possible fragmentation pathway of M11 in HRMS/MS spectrum

Figure S6 the possible fragmentation pathway of M12 in HRMS/MS spectrum

Figure S7	the possible fragmentation pathway of M13 in HRMS/MS spectrum

Figure S8	the possible fragmentation pathway of M14 in HRMS/MS spectrum

Figure S9	the possible fragmentation pathway of M15 in HRMS/MS spectrum

Figure S10	the possible fragmentation pathway of M16 in HRMS/MS spectrum
